# Longitudinal changes in hippocampal morphology before and after temporal lobe epilepsy surgery

**DOI:** 10.1093/braincomms/fcaf416

**Published:** 2025-10-24

**Authors:** Matus Velicky Buecheler, Richard Zubal, Robert Terziev, Andrew W McEvoy, Jane de Tisi, Anna Miserocchi, Sjoerd B Vos, Kai Michael Schubert, Christian R Baumann, John S Duncan, Matthias J Koepp, Marian Galovic

**Affiliations:** Department of Neurology, Clinical Neuroscience Centre, University Hospital and University of Zurich, Zurich 8091, Switzerland; Department of Neuroradiology, Inselspital, Bern University Hospital and University of Bern, Bern 3010, Switzerland; Department of Neurology, Clinical Neuroscience Centre, University Hospital and University of Zurich, Zurich 8091, Switzerland; Department of Clinical and Experimental Epilepsy, UCL Queen Square Institute of Neurology, London, London WC1N 3BG, United Kingdom; Department of Clinical and Experimental Epilepsy, UCL Queen Square Institute of Neurology, London, London WC1N 3BG, United Kingdom; Department of Clinical and Experimental Epilepsy, UCL Queen Square Institute of Neurology, London, London WC1N 3BG, United Kingdom; Department of Clinical and Experimental Epilepsy, UCL Queen Square Institute of Neurology, London, London WC1N 3BG, United Kingdom; Department of Computer Science, Centre for Medical Image Computing, University College London, London NW1 2AE, United Kingdom; Western Australia National Imaging Facility Node, The University of Western Australia, Nedlands 6009, Australia; Department of Neurology, Clinical Neuroscience Centre, University Hospital and University of Zurich, Zurich 8091, Switzerland; Department of Neurology, Clinical Neuroscience Centre, University Hospital and University of Zurich, Zurich 8091, Switzerland; Department of Clinical and Experimental Epilepsy, UCL Queen Square Institute of Neurology, London, London WC1N 3BG, United Kingdom; MRI Unit, Epilepsy Society, Chalfont St Peter, Buckinghamshire SL9 0RJ, United Kingdom; Department of Clinical and Experimental Epilepsy, UCL Queen Square Institute of Neurology, London, London WC1N 3BG, United Kingdom; MRI Unit, Epilepsy Society, Chalfont St Peter, Buckinghamshire SL9 0RJ, United Kingdom; Department of Neurology, Clinical Neuroscience Centre, University Hospital and University of Zurich, Zurich 8091, Switzerland; Department of Clinical and Experimental Epilepsy, UCL Queen Square Institute of Neurology, London, London WC1N 3BG, United Kingdom; MRI Unit, Epilepsy Society, Chalfont St Peter, Buckinghamshire SL9 0RJ, United Kingdom

**Keywords:** epilepsy, seizures, hippocampus, MRI, surgery

## Abstract

Temporal lobe epilepsy (TLE) is associated with progressive neocortical thinning that may be prevented by resective surgery, but little knowledge exists on dynamic changes in the hippocampus. Here, we assessed progressive morphological changes of the hippocampus before and after TLE surgery. In this longitudinal case-control neuroimaging study, we included patients with unilateral drug-resistant TLE before (main cohort *n* = 23; replication cohort *n* = 81) or after (*n* = 54) anterior temporal lobe resection and healthy volunteers (*n* = 120) matched for age and sex. We compared hippocampal volumes and surface shape morphology on paired scans between groups using linear mixed effects models. We did not find accelerated atrophy of the ipsilateral hippocampus in chronic presurgical TLE (−2.1 µl/year) compared to normal aging (−3.6 µl/year, *P* = 0.78). In contrast, there was progressive hypertrophy of the contralateral hippocampus before epilepsy surgery (+1.4 µl/year) compared to normal aging (−4.1 µl/year), which was further accelerated after surgery (+4.0 µl/year, *P* = 0.01). We validated these findings in the replication cohort (*n* = 81). Factors contributing to contralateral hippocampal hypertrophy included left-sided epilepsy lateralization, ipsilateral hippocampal sclerosis, and visual memory encoding deficits before or after surgery. Conversely, a history of focal to bilateral tonic clonic seizures was associated with accelerated pre- and postsurgical atrophy of the contralateral hippocampus. Refractory TLE is associated with progressive hypertrophy of the contralateral hippocampus before and after resective surgery. These contralateral changes may represent compensatory plasticity that is related to pre- and postoperative disturbances in cognition, mainly affecting visual memory. In contrast to progressive cortical thinning, the ipsilateral hippocampus does not show additional atrophy in chronic TLE.

## Teaser

Velicky Buecheler *et al*. have studied accelerated growth or shrinkage of the hippocampus in people with epilepsy and concluded that there is progressive growth of the hippocampus on the side opposite to the epileptic focus. This growth may relate to compensation of cognitive deficits.

## Graphical Abstract

**Graphical Abstract:**
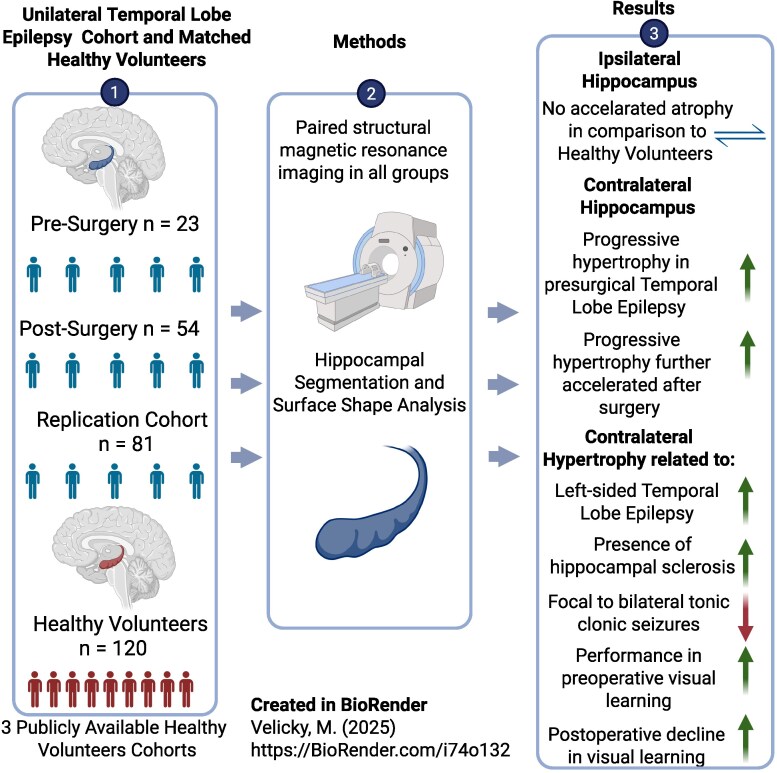

## Introduction

Temporal lobe epilepsy (TLE) is one of the most common epilepsy syndromes in adults.^1^ TLE is frequently drug-resistant and is commonly treated surgically. The most common underlying pathology of TLE is hippocampal sclerosis (HS), which can be reliably visualized using magnetic resonance imaging (MRI).^2,3^

Recent studies suggested that TLE may be a dynamic, rather than a static disease.^4,5^ Longitudinal MRI studies found progressive neocortical atrophy in TLE.^4,6,7^ Accelerated neocortical thinning may be effectively interrupted by successful epilepsy surgery, particularly in those who remain completely seizure-free following surgery.^5^

Longitudinal imaging data on progressive hippocampal changes are less robust. Some, but not all, longitudinal studies found progressive atrophy of the hippocampus.^8,9^ These studies had, however, lacked a control group to adjust for the effects of normal aging. A large longitudinal population-based study did not find progressive hippocampal atrophy in newly-diagnosed epilepsy.^10^ The lack of consensus in previous literature may be a result of underpowered studies, or because there is significant variation in hippocampal shape abnormalities in TLE that may be missed in simple volumetric analyses.^13,14^

In this longitudinal case-control neuroimaging study, we assessed hippocampal volumes and surface shapes in patients with chronic drug-refractory unilateral TLE before and after anterior temporal lobe resection (ATLR). We aimed to determine whether there are progressive morphological changes in the hippocampus before or after resective surgery. In light of previous research,^4,5^ our hypothesis was bilateral progressive hippocampal atrophy before surgery that would be halted by successful epilepsy surgery.

## Materials and methods

### Participants

We identified consecutive patients with medically refractory TLE undergoing standard anterior temporal lobe resection at the National Hospital for Neurology and Neurosurgery (NHNN) in London, UK, from an ongoing prospective cohort study of long-term outcome after epilepsy surgery.^15^ We largely followed the approach described in our previous study focusing on cortical morphology.^5^ We included patients with TLE who (i) had serial high-resolution T1-weighted MRI scans at least 6 months apart performed on the same scanner before (presurgical group) or after (postsurgical group) surgery, (ii) have undergone resective surgery by the same neurosurgeon (A.W.M.), and (iii) have been followed-up postoperatively for at least 1 year. The minimum 6-month interval in between the serial scans was set to allow the subtle time-dependent morphological changes to be detected using longitudinal MRI. We excluded participants with a history of a neurodegenerative disease, stroke, white matter lesions or other relevant active neurological disorder. Participants with lesions other than hippocampal sclerosis (HS) affecting the hippocampal morphology as well as scans of insufficient quality (due to technical aspects of movement artefacts) were also excluded. Lesions not affecting the hippocampus were not excluded.

The diagnosis of TLE was made by a multidisciplinary epilepsy team evaluation considering clinical history, neurological examination, seizure semiology, long-term video- electroencephalography telemetry, MRI and neuropsychological and psychiatric assessments. Postoperative seizure freedom was defined as absence of seizures or auras throughout the postsurgical follow-up with exception of seizures within the first week after surgery (ILAE Class 1a). The size of the postsurgical hippocampal remnant was measured in the sagittal plane from the most anterior to the most posterior voxel along the hippocampal anterior-posterior axis.^35,36^

To validate our presurgical findings we additionally included independent data of patients with chronic unilateral TLE under follow-up at the NHNN who had serial preoperative scans but either did not have surgery or did not have serial postoperative scans. Participants in this ‘replication cohort’ followed the exclusion criteria described above and were longitudinally scanned on the same scanner using the same sequences as the main study cohort.^4^

The data in people with TLE were compared with 120 sex- and age-matched healthy volunteers from three publicly available anonymized cohorts who had two 3D-T1-weighted MRI scans on the same 3T scanner at least 6 months apart.^16-18^ The detailed healthy volunteer cohorts are described in the online supplement **(**Supplemental Table 1**)**.

The graphical flowchart of the study is shown in Supplemental Fig. 1. MRI Acquisition parameters and details of the neurosurgical procedure are described in the online supplement.

### Standard protocol approvals, registrations, and patient consents

The patient dataset comprised de-identified previously collected routine clinical information, without the need for individual consent, as approved by the UK Health Research Authority and local research ethics committee (22/SC/0016).

### Hippocampal segmentation and volumetry

We used Hipposeg (http://niftyweb.cs.ucl.ac.uk/program.php?p=HIPPOSEG)^14,19^ to automatically segment the ipsilateral and contralateral hippocampi before surgery and the contralateral hippocampi after surgery in people with TLE.^20^ Hipposeg was developed specifically for the segmentation of hippocampi in people with epilepsy with comparable variability as seen between expert human rater while being robust to atrophic hippocampi. We also segmented both hippocampi of healthy volunteers using the same procedure. The ipsilateral hippocampus was not segmented postsurgically because it has been partially resected during surgery.

Assumptions for our methods were that (i) the relationship between volume change and age is linear, (ii) Hipposeg provides comparable accuracy when segmenting healthy and sclerotic hippocampi, and (iii) the measured hippocampal volume is not or only minimally affected by peri-ictal changes such as oedema. The advantages of the method used for hippocampal segmentation have been described elsewhere in detail.^19^

All anonymized hippocampal masks were visually inspected and, if necessary, adjusted by an experienced investigator (M.V.B.) who was blinded to group allocation. This procedure previously showed a high intra-rater (0.98 ± 0.01) and inter-rater (0.96 ± 0.02) reliability.^14^ Additionally, we visually inspected and, if necessary, corrected outliers (≥ 2 standard deviations from group mean) from hippocampal volumes and volume changes. To account for differences in head size we corrected hippocampal volumes for total intracranial volume (TIV) using previously described formulas.^19^ We used the mean TIV across all scans within an individual subject to reduce the impact of TIV measurement variability on the changes of corrected hippocampal volumes.

### Hippocampal surface shape analysis

To assess ipsilateral and contralateral hippocampi separately, we flipped the hippocampal volumes and segmentations of people with right TLE and the same proportion of randomly selected healthy volunteers. In other words, in TLE and healthy volunteers the unflipped left and flipped right hippocampi were considered ‘ipsilateral,’ and vice versa. The hippocampal segmentations were then converted into 3D surface meshes with 1002 points using a spherical harmonics point distribution model (SPHARM-PDM) implemented in the Slicer SALT extension in 3D Slicer following minimal smoothing to ensure spherical topology.^13^ We aligned surfaces to a mean hippocampal template mesh generated from a normative database of healthy volunteers and visually checked for both surface mesh and alignment failures.

We parametrized surface displacement by calculating the shortest perpendicular distance between each point on an individual’s hippocampal surface mesh and the mean template surface.

We conceptualized atrophy as inward surface displacement (negative displacement value, blue colours) and hypertrophy as outward surface displacement (positive displacement value, red colours). As an illustration and approximation of hippocampal subregions, we drew heuristic boundaries on hippocampal surfaces, based on surface projections of histological data.^13^

### Neuropsychological evaluation

Data obtained during a standardized neuropsychological evaluation were available in 15/23 TLE patients before and in 46/54 after surgery. Verbal and visual learning were evaluated preoperatively and 1 year postoperatively using the list learning and design learning tasks respectively included in the Adult Memory and Information Processing Battery.^21^ Change in neuropsychological performance 1 year after epilepsy surgery in the postsurgical cohort was quantified as the difference in pre- and post-surgical memory z-scores. The verbal and performance intelligence quotients (IQ) were assessed preoperatively using the Wechsler Adult Intelligence Scale. Detailed neurocognitive results are summarized in the online supplement **(**Supplemental Table 2**)**.

### Statistical analysis

Categorical variables are displayed as *n* (%) and were analysed with Fisher’s exact test. Numerical variables are displayed as mean ± standard deviation (SD) or median and interquartile range (IQR) when more appropriate and were assessed with one-way analysis of variance. Calculations were carried out in SPSS (IBM Corp, Version 25.0).

We compared changes in the volume and surface shape of the presurgical ipsilateral hippocampi and the pre- and postsurgical contralateral hippocampi with aging-related hippocampal changes in healthy volunteers. Volumetric and point-wise hippocampal data were analysed using SurfStat (http://www.math.mcgill.ca/keith/surfstat) within MATLAB (MathWorks). We fitted linear mixed-effects models, a flexible framework for longitudinal analysis of multiple repeated measurements per subject with irregular measurement intervals. To test for between-group differences in changes of hippocampal surface shape morphology over time, we evaluated an interaction effect between the group allocation and age at scan, correcting for a random effect of subject and fixed effects of the co-variates age at scan, sex, group, and TIV. With this approach, we were able to test for within-subject morphological changes over time while correcting for baseline demographic differences and for different inter-scan intervals. We also adjusted for the presence of ipsilateral sclerosis when directly comparing the pre- versus postsurgical TLE groups, because there was a non-significant trend towards more frequent hippocampal sclerosis in the postsurgical TLE cases (Table 1). Cognitive analyses were additionally adjusted for epilepsy lateralisation. Changes in hippocampal volumes, which were already corrected for head size, were analysed using the same models without additional adjustment for TIV. We report findings at *P* < 0.05 corrected for multiple comparisons using random field theory for non-isotropic images on a cluster level.^22^ Annualized hippocampal volume changes in each group were estimated as the predicted slope of the linear regression lines from the mixed effects models.

**Table 1:** Baseline characteristics of the main cohorts

	TLE presurgical *n* = 23	TLE postsurgical *n* = 54	Healthy Volunteers *n* = 120	*P*-Value
Sex				
Male	12 (52%)	20 (37%)	46 (38%)	0.45
Female	11 (48%)	34 (63%)	74 (62%)	
Age and intervals *(years)*				
Age at scan	38 (15)	42 (18)	28 (35)	0.59
Interval between scans	2.1 (1.7)	0.8 (0.4)	1.6 (1.2)	<0.001
Age at surgery	43 (15)	42 (18)	-	0.75
Duration of epilepsy at surgery	23 (19)	24 (25)	-	0.95
Age at seizure onset	14 (11)	11 (10)	-	0.76
Presurgical seizures				
Focal aware	9 (39%)	30 (55%)	-	0.22
Focal impaired awareness	22 (95%)	52 (96%)	-	1.00
Focal to bilateral tonic clonic	18 (78%)	43 (79%)	-	0.56
Side of surgery				
Right	9 (39%)	24 (44%)	-	0.80
Left	14 (61%)	30 (56%)	-	
Pathology				
Hippocampal sclerosis	13 (56%)	41 (76%)	-	0.11
Dysembryoplastic neuroepithelial tumour	3 (13%)	4 (7%)	-	0.35
Cavernoma	0 (0%)	2 (3%)	-	0.49
Other	7 (30%)	9 (17%)	-	0.15
Surgical outcome				
Seizure free after surgery (Class Ia)	9 (39%)	22 (41%)	-	0.38
Other				
Number of ASMs at surgery	2 (1)	2 (1)	-	0.29
History of a precipitating injury	2 (8%)	4 (7%)	-	0.58
History of febrile convulsions	2 (8%)	7 (13%)	-	0.46
History of depression	9 (39%)	19 (35%)	-	0.47
History of psychosis	3 (13%)	3 (5%)	-	0.25
History of anxiety disorder	2 (8%)	9 (17%)	-	0.23

Data displayed as *n* (%) or median (IQR). Data analysed with Fisher’s exact test for nominal variables or with one-way ANOVA for scalar variables. ASM, antiseizure medication; IQR, interquartile range.

We performed several secondary analyses. First, we validated the findings in a separate replication TLE cohort with presurgical longitudinal imaging using the same models. Second, we performed regression analyses using the same modelling approach to examine the association between clinical variables (age at onset of epilepsy, epilepsy duration and lateralisation, presence of hippocampal sclerosis, volume of the ipsilateral hippocampus, presurgical seizure frequency, FBTCS before surgery, number of ASMs at surgery, ongoing seizures after surgery, and the size of the hippocampal remnant) and neuropsychological variables (preoperative verbal and visual learning, preoperative verbal and performance IQ, postoperative decline in verbal and visual learning) with the rate of contralateral hippocampal changes in TLE. These analyses were restricted to patients with TLE (i.e. not including healthy controls) and replaced group allocation in the statistical model with the variable of interest. Third, we compared pre- and postsurgical hippocampal morphology in the subgroup of patients (*n* = 8) who had both pre- and postsurgical paired scans. Finally, we repeated the main and neuropsychological analyses separately in the subgroups with left and right TLE.

## Results

We included 23 patients with TLE and paired scans before surgery (14 left TLE) and 54 patients with TLE and paired scans after surgery (30 left TLE). Eight patients had both pre- and postsurgical paired scans (i.e. 4 scans altogether per person). All patients with TLE underwent unilateral anterior temporal lobe resection. We compared the pre- and postsurgical groups with 120 healthy volunteers that were comparable for age and sex. Except for differences in interscan interval, there were no differences between the pre- and postsurgical groups in baseline characteristics (**Table 1**).

### Changes in hippocampal morphology before and after surgery

**Figure 1:**
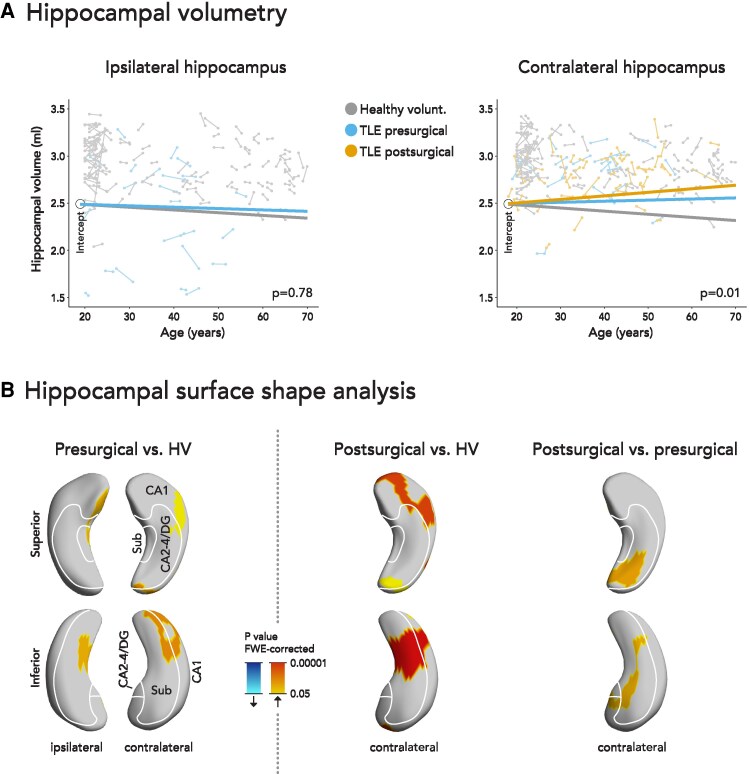
**Progressive morphological changes of the hippocampus in TLE before and after surgery.** (**A**) Changes of ipsilateral (left) and contralateral (right) hippocampal volumes in healthy volunteers (grey), pre- (blue) and postoperative (orange) TLE patients. Each scan is represented by a dot and scans corresponding to the same subject are connected by a thin line. The three thick lines are linear regression lines of mixed effects models and their slopes represent the estimated rate of hippocampal volume change in each group. We used linear mixed effects models in all participants (presurgical TLE, n = 23; postsurgical TLE, n = 54; healthy volunteers, n = 120). Although mixed effects models were ﬁtted with a variable intercept, for demonstration purposes we display the linear regression lines having the same intercept to improve legibility. (**B**) Subregional longitudinal changes in hippocampal morphology. We compared progressive changes in both hippocampi in presurgical TLE patients with healthy volunteers (left), the contralateral hippocampus in postsurgical TLE patients with healthy volunteers (middle), and the contralateral hippocampus in post- versus presurgical TLE patients (right). Blue clusters indicate significant progressive atrophy, red colours indicate significant progressive hypertrophy. We used linear mixed effects models in all participants (presurgical TLE, n = 23; postsurgical TLE, n = 54; healthy volunteers, n = 120). Signiﬁcant *P* values were thresholded to *P* < 0.05 corrected for multiple comparisons using familywise error (FWE) correction by random ﬁeld theory. The ipsi- and contralateral hippocampi are visualized from a superior and an inferior perspective. An approximation of major hippocampal subregional boundaries is overlaid on hippocampal surfaces. CA, cornu ammonis; DG, dentate gyrus; FWE, familywise error; HV, healthy volunteers; Sub, subiculum; TLE, temporal lobe epilepsy.

The overall volume of the ipsilateral hippocampus in presurgical chronic TLE did not change compared to normal aging in healthy volunteers (−2.1 ± 5.6 versus −3.6 ± 1.7 µl/year, degrees of freedom [df]= 277, *t* = 0.3, *P* = 0.78, Fig. 1A, Supplemental Fig. 2). Surface shape analysis detected an area in the mesial ipsilateral hippocampal body with reduced aging-related atrophy in presurgical TLE (70 points on the surface hippocampal map, *P* = 0.003, Fig. 1B).

**Figure 2:**
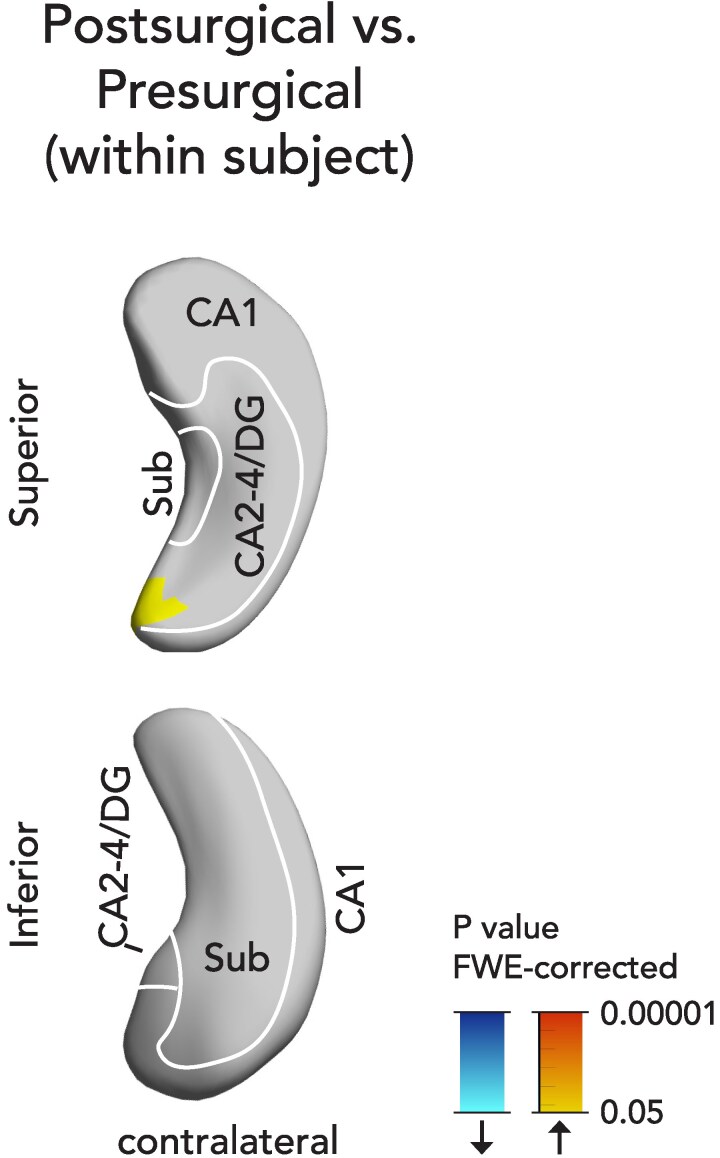
**Progressive morphological changes of the hippocampus in TLE before versus after surgery in those with both paired pre- and postsurgical scans.** The figure shows subregional longitudinal changes in hippocampal morphology. We compared progressive changes in the contralateral hippocampus in before surgery versus after surgery in TLE patients (n = 8) who had two scans before surgery and two scans after surgery. Blue clusters indicate significant progressive atrophy, yellow/red colours indicate significant progressive hypertrophy. Signiﬁcant *P* values calculated using a linear mixed effects model in 8 subjects were thresholded to *P* < 0.05 corrected for multiple comparisons using familywise error (FWE) correction by random ﬁeld theory. The ipsi- and contralateral hippocampi are visualized from a superior and an inferior perspective. An approximation of major hippocampal subregional boundaries is overlaid on hippocampal surfaces. CA, cornu ammonis; DG, dentate gyrus; FWE, familywise error; Sub, subiculum; TLE, temporal lobe epilepsy.

The contralateral hippocampus showed progressive hypertrophy in presurgical TLE (+1.4 ± 6.7 µl/year) compared to normal aging (−4.1 ± 2.6 µl/year), which was further accelerated after surgery (+4.0 ± 5.9 µl/year, df = 294, *t* = 3.0, *P* = 0.01, Fig. 1A, Supplemental Fig. 2). Presurgical hypertrophy of the contralateral hippocampus was confirmed using surface shape analysis in the inferior tail (84 points, *P* < 0.001) and lateral head/body (45 points, *P* = 0.03, Fig. 1B). After surgery, the contralateral hippocampus showed accelerated hypertrophy compared to normal aging (inferior tail, 112 points, *P* < 0.001; superior head, 103 points, *P* < 0.001; superior tail, 42 points, *P* = 0.03) and compared to preoperative TLE (superior tail, 68 points, *P* = 0.002; inferior head/body, 67 points, *P* = 0.003). In a within-subject analysis the results were similar (see Fig. 2). The results were similar when splitting the groups into left and right TLE, whereas contralateral hypertrophy was more pronounced in left compared to right TLE (Supplemental Fig. 3).

### Changes in hippocampal morphology in the replication cohort

To validate our findings, we replicated the presurgical results in an independent cohort of 81 patients with chronic unilateral TLE. Their baseline characteristics are displayed in **Table 2**.

**Table 2:** Baseline characteristics of the replication cohort of patients with unilateral TLE (n = 81)

	Replication TLE cohort *n* = 81
Sex	
Male	43 (53%)
Female	38 (47%)
Age *(years)*	
Age at scan	37 (17)
Interval between scans	2.3 (2.5)
Duration of epilepsy	25 (26)
Epilepsy lateralisation	
right	35 (43%)
left	46 (57%)
Other	
Hippocampal sclerosis	28 (35%)
Number of ASM	2 (1)

Data displayed as n (%) or median (IQR). ASM, antiseizure medication.

**Figure 3:**
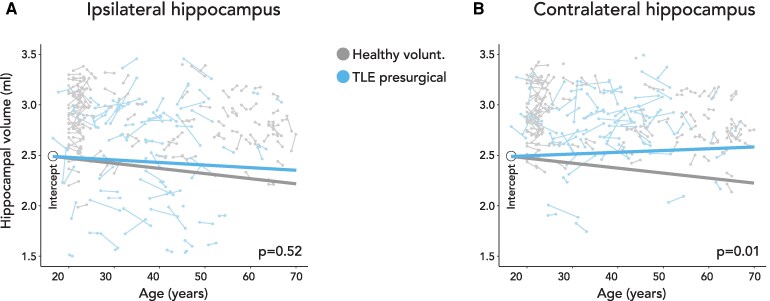
**Progressive morphological changes of the hippocampus in the replication cohort with unilateral TLE before surgery.** The figure shows changes of ipsilateral (**A**) and contralateral (**B**) hippocampal volumes in healthy volunteers (grey) and the replication cohort of unilateral preoperative TLE patients (blue). Each scan is represented by a dot and scans corresponding to the same subject are connected by a thin line. The three thick lines are linear regression lines of mixed effects models and their slopes represent the estimated rate of hippocampal volume change in each group. We used linear mixed effects models in all participants (replication TLE cohort, n = 81; healthy volunteers, n = 120). Although mixed effects models were ﬁtted with a variable intercept, for demonstration purposes the linear regression lines are displayed having the same intercept to improve legibility. TLE, temporal lobe epilepsy.

The overall volume of the ipsilateral hippocampus in the replication cohort did not change presurgically compared to normal aging in healthy volunteers (−2.8 ± 4.5 versus −5.3 ± 2.2 µl/year, df = 350, *t* = 0.6, *P* = 0.52, Fig. 3A). The contralateral hippocampus in presurgical TLE showed progressive hypertrophy compared to normal aging in healthy volunteers (+2.2 ± 3.2 versus 5.0 ± 1.6 µl/year, df = 378, *t* = 2.6, *P* = 0.01, Fig. 3B).

### Factors contributing to changes of contralateral hippocampal morphology

**Figure 4:**
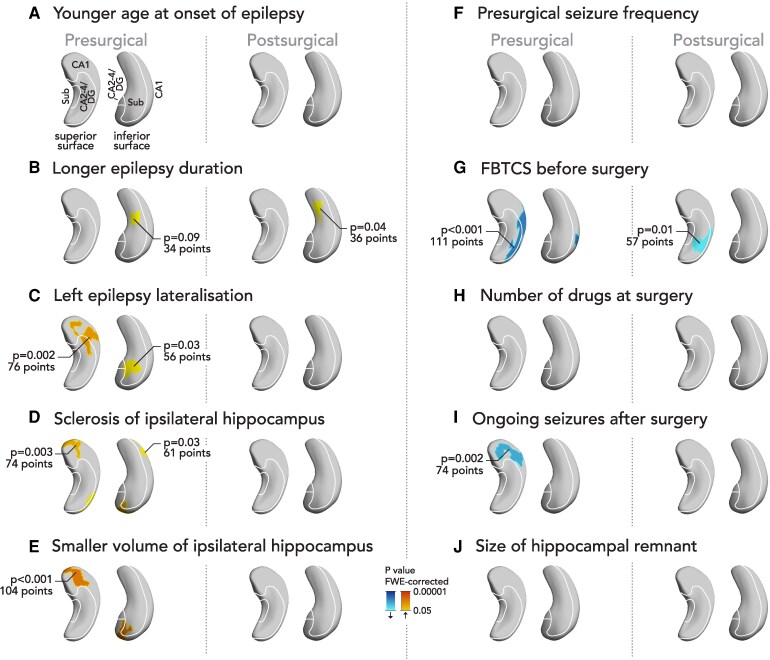
**Longitudinal changes of contralateral hippocampal morphology and their association with clinical variables.** We correlated the subregional longitudinal changes in contralateral hippocampal morphology before and after epilepsy surgery with clinical variables. We assessed the association with younger age at epilepsy onset (**A**), longer epilepsy duration (**B**), left lateralisation of the epileptic focus (**C**), sclerosis of the ipsilateral hippocampus (**D**), smaller volume of the ipsilateral hippocampus (**E**), presurgical seizure frequency (**F**), presurgical focal to bilateral tonic clonic seizures (FBTCS, **G**), number of antiseizure medications at surgery (**H**), ongoing seizures after epilepsy surgery (**I**) and the size of the hippocampal remnant (**J**). Blue clusters indicate significantly less progressive hypertrophy, red colours indicate significantly more progressive hypertrophy. We used linear mixed effects models in 23 presurgical and 54 postsurgical TLE patients. Significant *P* values were thresholded to *P* < 0.05 corrected for multiple comparisons using familywise error (FWE) correction by random ﬁeld theory. The contralateral hippocampi are visualized from a superior and an inferior perspective. An approximation of major hippocampal subregional boundaries is overlaid on hippocampal surfaces. CA, cornu ammonis; DG, dentate gyrus; FWE, familywise error; Sub, subiculum.

We evaluated factors that may contribute to pre- or postsurgical hypertrophy of the contralateral hippocampus in the main cohort (Fig. 4). Larger presurgical hypertrophy of the contralateral hippocampus was associated with a longer disease duration (Fig. 4B; trend presurgically, *P* = 0.09 and significant postsurgically, *P* = 0.04), left-sided lateralisation of the epileptic focus (Fig. 4C, *P* = 0.002 and *P* = 0.003), presence of ipsilateral hippocampal sclerosis (Fig. 4D, *P* = 0.003 and *P* = 0.03), and a smaller volume of the ipsilateral hippocampus (Fig. 4E, *P* < 0.001). Presurgical focal to bilateral tonic clonic seizures (FBTCS) were associated with *less* progressive hypertrophy of the contralateral hippocampus before and after surgery (Fig. 4G, *P* < 0.001 and *P* = 0.01 respectively). Progressive atrophy of the contralateral hippocampal head was associated with ongoing seizures after epilepsy surgery (Fig. 4I, *P* = 0.002). There was no association with age at epilepsy onset (Fig. 4A), presurgical seizure frequency (Fig. 4F), number of antiseizure medications (ASMs) at surgery (Fig. 4H), and the size of the postsurgical hippocampal remnant (Fig. 4J).

### Neuropsychological testing and changes to contralateral hippocampal morphology

**Figure 5:**
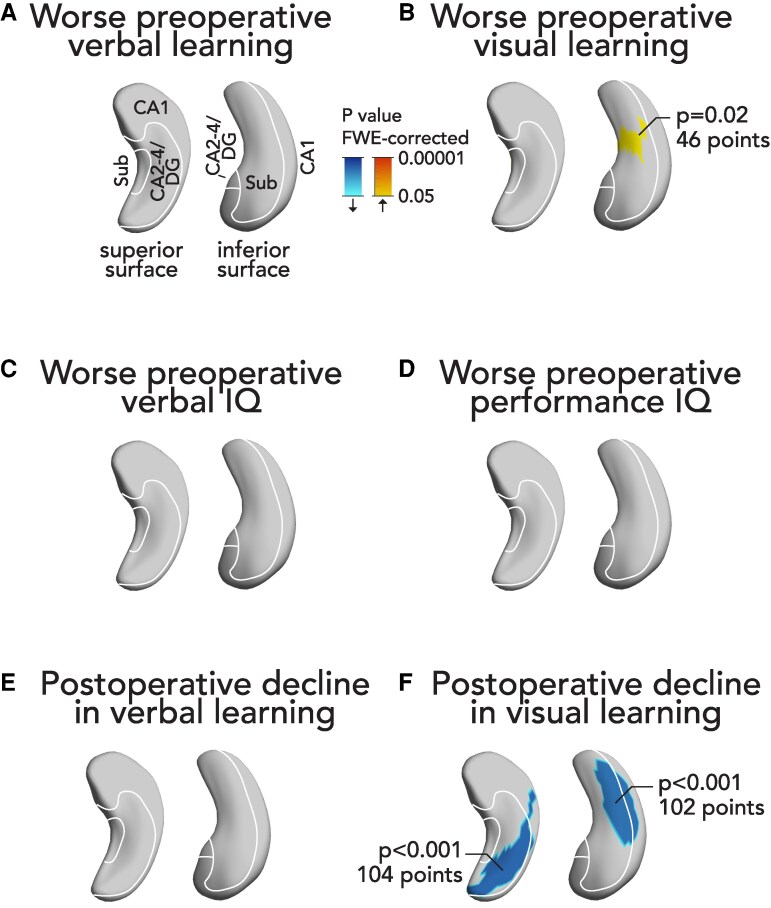
**Longitudinal changes of contralateral hippocampal morphology and their association with neurocognitive testing results.** We correlated the subregional longitudinal changes in contralateral hippocampal morphology with neurocognitive testing results. We assessed the association of presurgical hippocampal changes with worse preoperative verbal (**A**) or visual (**B**) learning, verbal IQ (**C**), and performance IQ (**D**). We also assessed the association of postsurgical hippocampal changes with postoperative decline in verbal (**E**) or visual (**F**) learning. Blue clusters indicate significantly less progressive hypertrophy, red colours indicate significantly more progressive hypertrophy. We used linear mixed effects models in 23 presurgical and 54 postsurgical TLE patients. Significant *P* values were thresholded to *P* < 0.05 corrected for multiple comparisons using familywise error (FWE) correction by random ﬁeld theory. The contralateral hippocampi are visualized from a superior and an inferior perspective. An approximation of major hippocampal subregional boundaries is overlaid on hippocampal surfaces. CA, cornu ammonis; DG, dentate gyrus; FWE, familywise error; IQ, intelligence quotient; Sub, subiculum.

Worse preoperative visual learning was associated with more progressive hypertrophy of the contralateral hippocampus before surgery in the main cohort (Fig. 5B). Preoperative verbal learning (Fig. 5A), verbal IQ (Fig. 5C), and performance IQ (Fig. 5D) were not associated with contralateral hippocampal morphological changes.

After epilepsy surgery, postoperative decline of visual learning was associated with less hypertrophy of the contralateral hippocampus in the main cohort (Fig. 5F). Postoperative decline of verbal learning (Fig. 5E) did not correlate with contralateral hippocampal surface shape.

We performed secondary analyses by examining these associations separately in left and right TLE (Supplemental Fig. 4). The association with worse preoperative and postoperative visual learning was replicated in left TLE but not in right TLE, which may reflect sample size differences and consequently reduced statistical power.

## Discussion

We assessed the longitudinal changes of hippocampal morphology before (ipsi- and contralateral hippocampi) and after (contralateral hippocampi) anterior temporal lobe resections in a well-characterized cohort of patients with chronic drug-refractory unilateral TLE and compared these to matched healthy volunteers. We did not find accelerated atrophy of the ipsilateral hippocampus in chronic TLE in our presurgical cohort. In contrast, the contralateral hippocampus showed progressive hypertrophy before surgery that was further accelerated in the postsurgical cohort. Factors contributing to contralateral hippocampal hypertrophy included longer disease duration, left-sided epilepsy lateralisation, presence of ipsilateral hippocampal sclerosis, lower ipsilateral hippocampal volume, and worse visual memory. We demonstrated the main findings of our study using two separate methods (volumetry and surface shape analysis) and validated them in a larger independent replication cohort of unilateral presurgical TLE.

### Ipsilateral hippocampus

In chronic drug-refractory TLE (mean disease duration 26 ± 13 years), we did not detect progressive atrophy of the hippocampus ipsilateral to the epileptic focus. This is in contrast with the finding of widespread and bilateral progressive cortical atrophy in previous studies performed on the same cohorts.^4,5^ These results suggest that hippocampal atrophy occurs early during the course of TLE, whereas cortical atrophy is progressive even at chronic disease stages. This notion has recently been confirmed in an event-based modelling study showing that hippocampal atrophy occurs at the earliest disease stage in TLE and is later followed by thalamic and neocortical atrophy.^23^ The development of hippocampal atrophy may be additionally driven by genetic or familial factors, as suggested by a study that found hippocampal volume reductions in unaffected siblings of patients with TLE.^11^ On the other hand, cortical atrophy seems to be acquired during the course of TLE because a genetic contribution could not be detected in unaffected siblings of patients with TLE.^11,12^

The overall rate of ipsilateral hippocampal atrophy in patients with TLE was not significantly different from normal aging (Figs. 1A, 2). Surface shape analysis detected a small area with slower atrophy than expected during normal aging (Fig. 1B). This could be explained by a reduced propensity of some hippocampal subregions to undergo additional aging-related atrophy, if they have been previously damaged by epilepsy. One potential explanation for our results is that ipsilateral hippocampal sclerosis may be occurring during early disease stages and that there is little additional atrophy in chronic TLE.

### Contralateral hippocampus

We demonstrated progressive hypertrophy of the contralateral hippocampus before and after epilepsy surgery using volumetry (Fig. 1A) and surface shape analysis (Fig. 1B) in the main cohort and in the replication cohort (Fig. 3). The progressive changes in the contralateral hippocampus may either reflect small growth of the hippocampus or a lack of aging related atrophy compared to healthy controls. We used the term hypertrophy although we cannot differentiate between these two explanations. For the sake of simplicity, we use the term hypertrophy instead of ‘progressive volume increase’ and atrophy instead of ‘progressive volume reduction’, although we do not provide neuropathological findings. Contralateral hypertrophy was more accelerated in the post-surgical compared to the presurgical cases. Consistent with our findings, one previous study found relative postsurgical increases in grey matter concentration, particularly in seizure-free cases and in the hemisphere contralateral to the resection.^24^

One potential explanation for our results is that contralateral hippocampal hypertrophy may represent plasticity to compensate for ipsilateral hippocampal damage. Contralateral hypertrophy was linked to morphological abnormalities of the ipsilateral hippocampus and was more pronounced if the ipsilateral hippocampus was sclerotic (Fig. 4D) or had a small volume (Fig. 4E). This also suggests that TLE without hippocampal changes, e.g. due to a neocortical lesion, has a smaller impact on contralateral hypertrophy. There was more contralateral hypertrophy in left TLE (Fig. 4C, Supplemental Fig. 3) which could be linked to the potential for larger network disruption if epilepsy affects the left, usually language-dominant, hemisphere. The acceleration of hippocampal hypertrophy after surgery could be interpreted as an inefficient or unsuccessful attempt of the brain to functionally compensate the resection of the ipsilateral hippocampus. Some results (Fig. 4B) suggest that contralateral hippocampal hypertrophy may be slightly accelerated in those with longer disease duration. We also observed that FBTCS were associated with reduced contralateral hypertrophy (Fig. 4G). A possible explanation is that FBTCS cause more extensive network disruption, which may interfere with compensatory mechanisms in the contralateral hemisphere and thereby limit hypertrophy.

The relationship of contralateral hypertrophy with cognitive findings additionally underscores the notion that hypertrophy may represent compensatory plastic changes. Contralateral hypertrophy mainly affecting the inferior medial hippocampal surface was related to worse visual memory before surgery (Fig. 5B). This is in line with previous findings that highlighted the relevance of the inferior medial hippocampal surface for visual memory encoding.^13^ However, contralateral hippocampal hypertrophy did not seem to completely compensate for the presurgical deficits. In other words, patients who had visual memory deficits before surgery may increasingly engage the contralateral hippocampus that may lead to morphological changes, but this does not seem to be fully sufficient to overcome such memory deficits. After surgery, decline of visual learning was associated with less contralateral hypertrophy (Fig. 5F). In other words, patients who effectively compensated for postsurgical deficits in visual memory encoding had more hypertrophy of the contralateral hippocampus.

Taken together, these findings point towards a compensatory role of contralateral hippocampal hypertrophy for visual memory deficits before and after epilepsy surgery and possibly indicate inefficient compensation of presurgical visual memory deficits. These findings appeared to be mostly driven by patients with left TLE, which could be explained by differences in statistical power or by differences in the underlying neuronal networks affected in left or right TLE.

The effects of use-dependent plasticity on brain structure have been prominently highlighted in London taxi drivers acquiring spatial navigation skills^25^ and people learning juggling.^26^ Our hypothesis of structural plasticity occurring in the contralateral hippocampus is directly supported by functional MRI studies using memory encoding paradigms. Patients with TLE and better visual memory engaged the mesial temporal lobe bilaterally, pointing towards an extension of the memory network into the contralateral mesial temporal lobe.^27^ Efficient compensation of memory deficits following surgery was related to engagement of the contralateral hippocampus.^28^

Contralateral hypertrophy in our study correlated with visual but not verbal memory findings and appeared to be driven by the left TLE group. This may be explained by the more symmetrical and bilateral representation of visual memory in both hippocampi, whereas verbal memory is more strictly lateralized in the language-dominant hemisphere.^13,27^ Thus, visual memory networks in left TLE may more efficiently harness the contralateral right hemisphere for plasticity whereas verbal memory may be less likely to benefit from contralateral plasticity.^27^

Other explanations for contralateral hippocampal hypertrophy are less likely. We did not observe effects of medication on contralateral hypertrophy. The notion that contralateral hypertrophy is linked to inflammation related to repeated seizures is unlikely because we did not see an association with seizure frequency. In contrast, FBTCS were associated with contralateral atrophy, which may point to contralateral hippocampal damage and atrophy induced by the extension of epileptic activity into the contralateral hemisphere. FBTCS are though to involve extensive and bilateral brain networks^29,30^ and were previously found to be related to more pronounced morphological abnormalities.^31^

Another interesting observation was the association of ongoing seizures after surgery with progressive contralateral hippocampal atrophy before surgery. This could point towards a more bilateral disease in these cases, thus making a unilateral resection less likely to render the patient seizure free. Similarly, previous studies found that structural abnormalities of the contralateral hippocampus were a predictor of poor surgical outcome.^32,33^

Two previous studies reported on a subset of patients with TLE that had bilateral hippocampal hypertrophy.^33,34^ This observation differs from our study because, in our study, we only included those with unilateral TLE and most of the subjects had unilateral hippocampal sclerosis.

### Methodological considerations

This study has limitations. First, our preoperative findings were observed in a cohort of patients with chronic refractory TLE scanned shortly before surgery and the findings should not be extrapolated to those with newly-diagnosed or early-stage TLE. Additionally, postoperative findings only apply to the first year after surgery and to standard anterior temporal lobe resections. Future studies should evaluate other surgical approaches and more long-term hippocampal changes. Second, data from patients with TLE and healthy volunteers were acquired on different 3T MRI scanners. As has been discussed in our previous studies,^4,5^ the statistical analyses focused on within-individual changes and all individuals were rescanned on the same equipment, minimizing the effect of between-cohort differences. Moreover, all groups were comparable for baseline characteristics and the statistical analyses were additionally adjusted for relevant covariates. Our previous analyses showed that the findings cannot be explained by a reduced sensitivity to detect structural changes in healthy volunteers or postsurgical patients compared with presurgical patients **(**Supplemental Table 3**)**.^4,5^ Nevertheless, despite our best efforts a residual confounding of the results by scanner differences cannot be completely excluded. Third, a limitation inherent in most epilepsy studies is the possible influence of ASM intake in patients compared with healthy volunteers. However, we did not find any association of ASM load with contralateral hippocampal hypertrophy before or after surgery (Fig. 4H). Additionally, medication withdrawal is usually not commenced during the first postoperative year at our centre. Thus, there were no differences in the pre- and postsurgical number of ASMs. Fourth, our epilepsy cohort was single-centre. Nevertheless, it is likely that the results are generalizable to other centres performing standard anterior temporal lobe resections, as established recommendations for this surgical procedure were followed. We replicated the results in a larger independent presurgical cohort of 81 people with unilateral TLE from our centre but did not perform external validation of our findings. Fifth, although flipping of hippocampal segmentations was performed in a comparable proportion of patients and controls, residual morphological differences between left and right hippocampi may have had an impact on our findings. Sixth, our cohorts were small and performing analyses in left and right TLE separately had low statistical power to detect significant findings. Nevertheless, our neuropsychological findings appear to be largely driven by the larger group with left TLE. Seventh, only 8 participants had both pre- and postsurgical paired MRI scans and most of our analyses relied on comparing groups of patients who had either paired presurgical or paired postsurgical scans. Lastly, causality cannot be confirmed in our study due to its retrospective design. Future studies should employ prospective designs that, albeit more challenging, may reduce bias and contribute to temporal clarity.

## Conclusion

Hypertrophy of the contralateral hippocampus occurs in chronic unilateral TLE and is further accelerated following resective surgery. This hypertrophy was related to deficits in visual memory and it may, potentially, represent a largely inefficient attempt of the contralateral hemisphere for cognitive compensation. This hypertrophy was related to deficits in visual memory and may represent a largely inefficient attempt of the contralateral hemisphere to provide cognitive compensation. If prospective studies combining neurocognitive testing with structural and functional MRI corroborate this interpretation, they could reinforce the importance of the contralateral hemisphere in mitigating neuropsychological deficits before and after epilepsy surgery.

This knowledge may then inform strategies to harness plasticity in the contralateral hemisphere to reduce memory deficits before (prehabilitation) and after (rehabilitation) epilepsy surgery. Hypothetically, memory-focused cognitive training that emphasizes visuospatial and scene-encoding strategies and engages contralateral mesial temporal networks may be particularly effective, especially in left TLE where effects were most pronounced. Experimentally, network-guided neuromodulation, such as stimulation of hippocampal-connected thalamic nodes (anterior or pulvinar nuclei) or direct hippocampal stimulation, might further enhance contralateral plasticity and holds potential to improve memory outcomes.

## Supplementary Material

fcaf416_Supplementary_Data

## Data Availability

The anonymized data are available upon reasonable request. The source MRI data were obtained clinically and the participants did not consent to sharing of their data with external investigators. No specific code was generated in this study.
