# Supplementary material for: Longitudinal changes in hippocampal morphology before and after temporal lobe epilepsy surgery
Source: Brain Commun. 2025 Oct 24;7(6):fcaf416. doi: 10.1093/braincomms/fcaf416 (PMC12603362; doi:10.1093/braincomms/fcaf416)
Supplement: fcaf416_Supplementary_Data [file fcaf416_supplementary_data.doc]

**Longitudinal changes in hippocampal morphology before and after temporal lobe epilepsy surgery**

*– ONLINE SUPPLEMENT –*

**1. Description of healthy volunteer cohorts**

The caveats regarding the use of publicly available longitudinal data of healthy volunteers have been described in the online supplement of our previous work^1^.

Analogous to our previous morphometric studies, we used longitudinal data from three publicly available cohorts of healthy volunteers aged between 20 to 70 years, having two 3T T1-weighted MRI scans on the same scanner at least 6 months apart.

**Supplemental Table 1:** *Healthy volunteer demographics by cohort*

|  | NMorphCH  (*n* = 24) | PPMI  (*n* = 48) | SLIM  (*n* = 53) |
| --- | --- | --- | --- |
| Sex |  |  |  |
| Female | 10 (42%) | 19 (40%) | 47 (89%) |
| Male | 14 (58%) | 29 (60%) | 6 (11%) |
| Age at baseline scan *(years)* | 32 ± 9 | 56 ± 9 | 21 ± 1 |
| Interval between scans *(years)* | 1.7 ± 0.4 | 1.2 ± 0.4 | 2.1 ± 0.6 |

**1.1 Neuromorphometry by Computer Algorithm Chicago (NMorphCH)**

Number of volunteers: 24

Reference for Dataset: <http://schizconnect.org>

Obtained through: SchizConnect

Reference for SchizConnect^2^: *Kogan A, Alpert K, Ambite JL, Marcus DS, Wang L. Northwestern University schizophrenia data sharing for SchizConnect: A longitudinal dataset for large-scale integration. Neuroimage 2016; 124: 1196–201.*

MR-acquisition: 3T Siemens TrioTim MRI scanner (Siemens Medical, Erlangen, Germany). A magnetization-prepared rapid gradient echo (MPRAGE) sequence was used to acquire high-resolution T1-weighted anatomical images (repetition time=2400 ms, echo time=3.16 ms, flip=8°, 256 x 256 matrix, 176 slices, slice thickness=1.0 mm, voxel size=1x1x1mm 3).

**1.2 Parkinson Progression Marker Initiative (PPMI)**

Number of volunteers: 48

Reference for Dataset^3^: *Parkinson Progression Marker Initiative. The Parkinson Progression Marker Initiative (PPMI). Progress in Neurobiology 2011; 95: 629-35.*

MR-acquisition: Scans used for this longitudinal cohort were acquired on 3T Siemens TrioTim or Verio MRI scanners (Siemens Medical, Erlangen, Germany). A magnetization-prepared rapid gradient echo (MPRAGE) sequence was used to acquire high-resolution T1-weighted anatomical images (repetition time=2300, echo time=2.98, flip angle=9°, 240 x 256 matrix, 160-192 slices, slice thickness=1.0 mm, voxel size=1x1x1mm 3). The T1 acquisition protocol followed ADNI-3 sequence parameter recommendations:

<http://adni.loni.usc.edu/wp-content/uploads/2017/07/ADNI3-MRI-protocols.pdf>

Detailed description of MR-acquisition protocol can be found in the PPMI MRI Technical Operations Manual:

<https://www.ppmi-info.org/sites/default/files/docs/archives/PPMI-MRI-Operations-Manual-V7.pdf>

**1.3 Southwest University Longitudinal Imaging Multimodal study (SLIM)**

Number of volunteers: 53

Reference for Dataset^4^: *Liu W, Wei D, Chen Q, et al. Longitudinal test-retest neuroimaging data from healthy young adults in southwest China. Sci Data 2017; 4: 170017*.

MR-acquisition: 3T Siemens Trio MRI scanner (Siemens Medical, Erlangen, Germany). A magnetization-prepared rapid gradient echo (MPRAGE) sequence was used to acquire high-resolution T1-weighted anatomical images (repetition time = 1900 ms, echo time = 2.52 ms, inversion time = 900 ms, flip angle = 9 degrees, resolution matrix = 256 x 256, slices = 176, thickness = 1.0 mm, voxel size=1x1x1mm 3).

Hippocampal masks of three healthy volunteers - 1 volunteer from the NMorphCH, 2 from the SLIM and PPMI cohorts - (n = 4) could not be analysed further due to technical problems, so the definite number of healhy volunteers analysed was 120.

**2. MRI Acquisition in epilepsy cohort**

MRI data of the TLE cohort were acquired between August 2004 and March 2013 on the same 3T MRI GE Signa HDx scanner (GE, Milwaukee, WI, USA), with the same MRI image sequence used for analysis: coronal T1W 3D inversion recovery fast spoiled gradient echo (IR-FSPGR) with repetition time / echo time / inversion time = 8.1 / 3.1 / 450 ms; field-of-view 187x240x240 mm; matrix 170x256x256, voxel dimensions 0.9x0.9x1.1mm.

**3. Neurosurgical procedure**

The same standard neurosurgical procedure has been used as described in our previous work^1^. It consisted of identifying the temporal horn entering from the collateral sulcus to minimize damage to the optic radiation and removing the temporal pole en bloc. This was followed by debulking of the amygdala, resection of the piriform cortex and en bloc resection of the hippocampus with a posterior resection margin at the midbrainstem level. The resection of the parahippocampal gyrus is also taken to the same level as the hippocampus. Typically, the anterior-posterior extent of the temporal lobe resection as measured from the temporal pole to the posterior margin of resection is 30% and 35% of the distance from the temporal pole to the occipital pole after left and right anterior temporal lobe resection, respectively. As surgery was performed by the same operator there was little variation of the temporal neocortical extent of the resection.

**4. Neuropsychology results**

**Supplemental Table 2:** *Neurocognitive results before and after surgery*

| **Neuropsychology before surgery*** | |
| --- | --- |
| Verbal IQ | 89.73 ± 11.43 |
| Performance IQ | 93.33 ± 9.48 |
| Verbal learning | 42.07 ± 10.10 |
| Visual learning | 25.60 ± 12.41 |
| **Neuropsychology 1 year after surgery**** | |
| Verbal learning | 37.67 ± 14.88 |
| Visual learning | 29.24 ± 7.56 |
| Change in verbal learning (z-score) | - 0.75 ± 1.18 |
| Change in visual learning (z-score) | - 0.11 ± 1.36 |

Data presented as mean ± standard deviation. * Data available in 15 cases in the presurgical TLE group; 3 data-points missing for performance IQ. ** Data available in 46 cases in the postsurgical TLE group; 1 data point missing for visual learning and change in visual learning.

**5. Supplemental Figure 1:** *Graphical flow chart of the study*


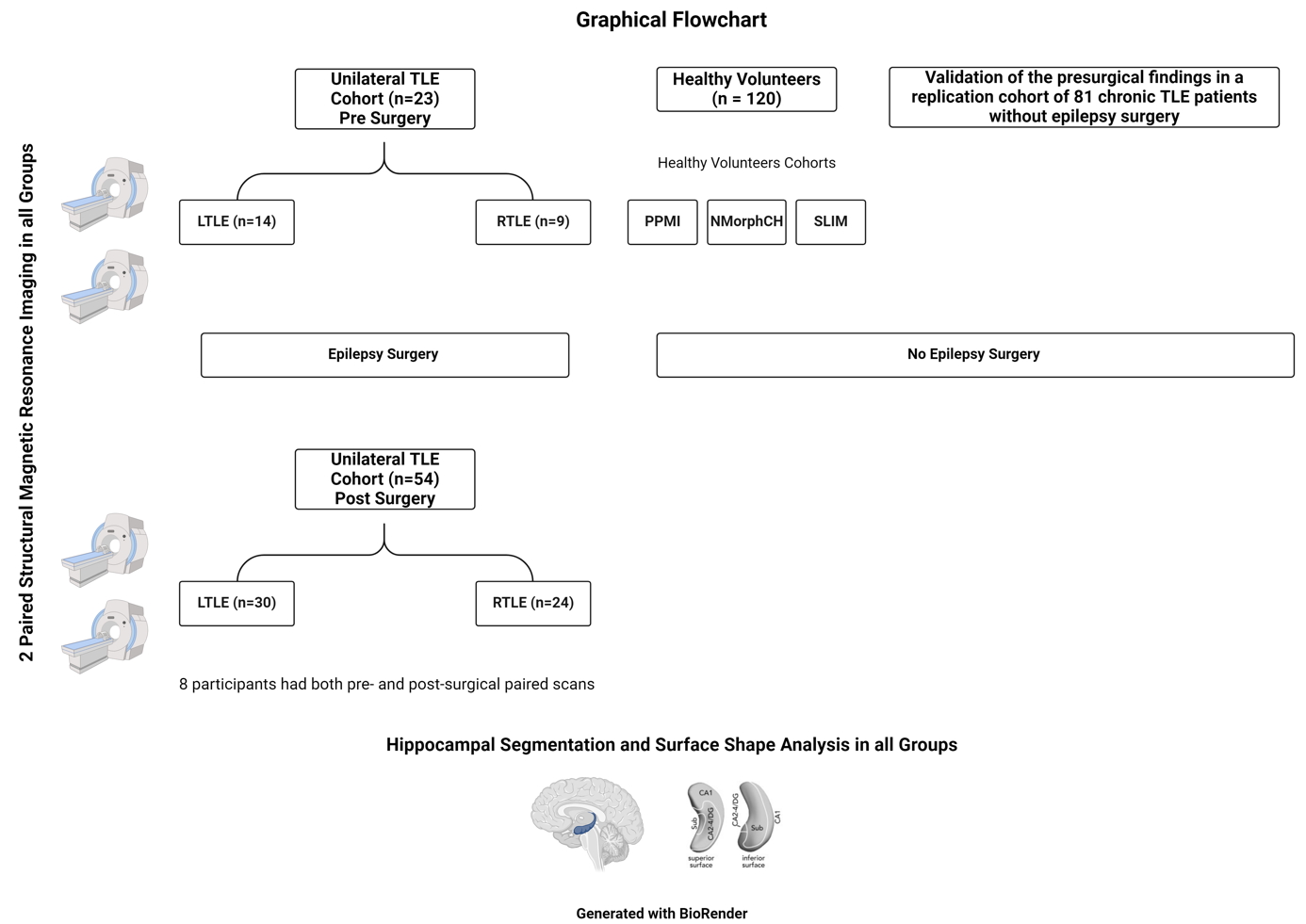


The figure shows the graphical flow-chart of the study. LTLE = left temporal lobe epilepsy; RTLE = right temporal lobe epilepsy; TLE = temporal lobe epilepsy.

Created in BioRender. Velicky, M. (2025) [*https://BioRender.com/c18y819*](https://biorender.com/c18y819)

**6. Supplemental Figure 2:** *Spaghetti plot of volumetric findings*


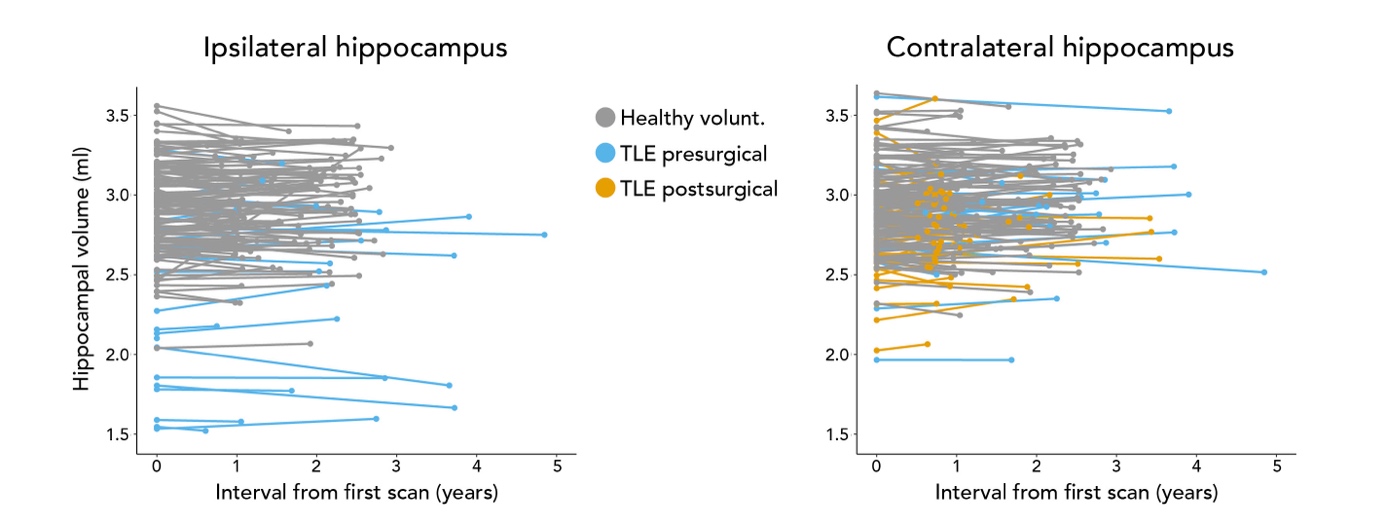


The figure shows changes in ipsilateral (left) and contralateral (right) hippocampal volumes in healthy volunteers (grey), pre- (blue) and postoperative (orange) TLE patients across the intervals between the baseline scans and repeat scans. The data is the same as in Fig. 1A in the main manuscript but the x-axis has been changed to provide an alternative visualization of the results. TLE = temporal lobe epilepsy.

**6. Supplemental Figure 3:** *Progressive morphological changes of the hippocampus in TLE before and after surgery, analysed separately for left and right TLE.*

The figure shows the rate of hippocampal volume change separately in left and right TLE. The panels show changes of ipsilateral (left) and contralateral (right) hippocampal volumes in healthy volunteers (grey), pre- (blue) and postoperative (orange) TLE patients. Each scan is represented by a dot and scans corresponding to the same subject are connected by a thin line. The three thick lines are linear regression lines of mixed effects models and their slopes represent the estimated rate of hippocampal volume. We used linear mixed effects models in all participants (presurgical TLE, left n=14 and right n=9; postsurgical TLE, left n=30 and right n=24; healthy volunteers, n=120) to calculate hippocampal volume change in each group. Although mixed effects models were ﬁtted with a variable intercept, for demonstration purposes we display the linear regression lines having the same intercept to improve legibility.

The results are similar to those in the main analyses (Fig. 1A) although there was a smaller power to detect significant findings due to a smaller sample size. The rate of ipsilateral hippocampal volume change did not differ from healthy volunteers in left and right TLE. Presurgical accelerated volume increase of the contralateral hippocampus was observed in left and not in right TLE, which is in line with Fig. 4C in the main manuscript. Postsurgical accelerated volume increase of the contralateral hippocampus was observed in left (p=0.02), but not in right (p=0.22) TLE. Some differences between left and right TLE may be explained by a larger sample size and, thus, higher statistical power to detect significant findings in left TLE. TLE = temporal lobe epilepsy.

**7. Supplemental Figure 4:** *Longitudinal changes of contralateral hippocampal morphology and their association with neurocognitive testing results, analyzed separately for left and right TLE.*


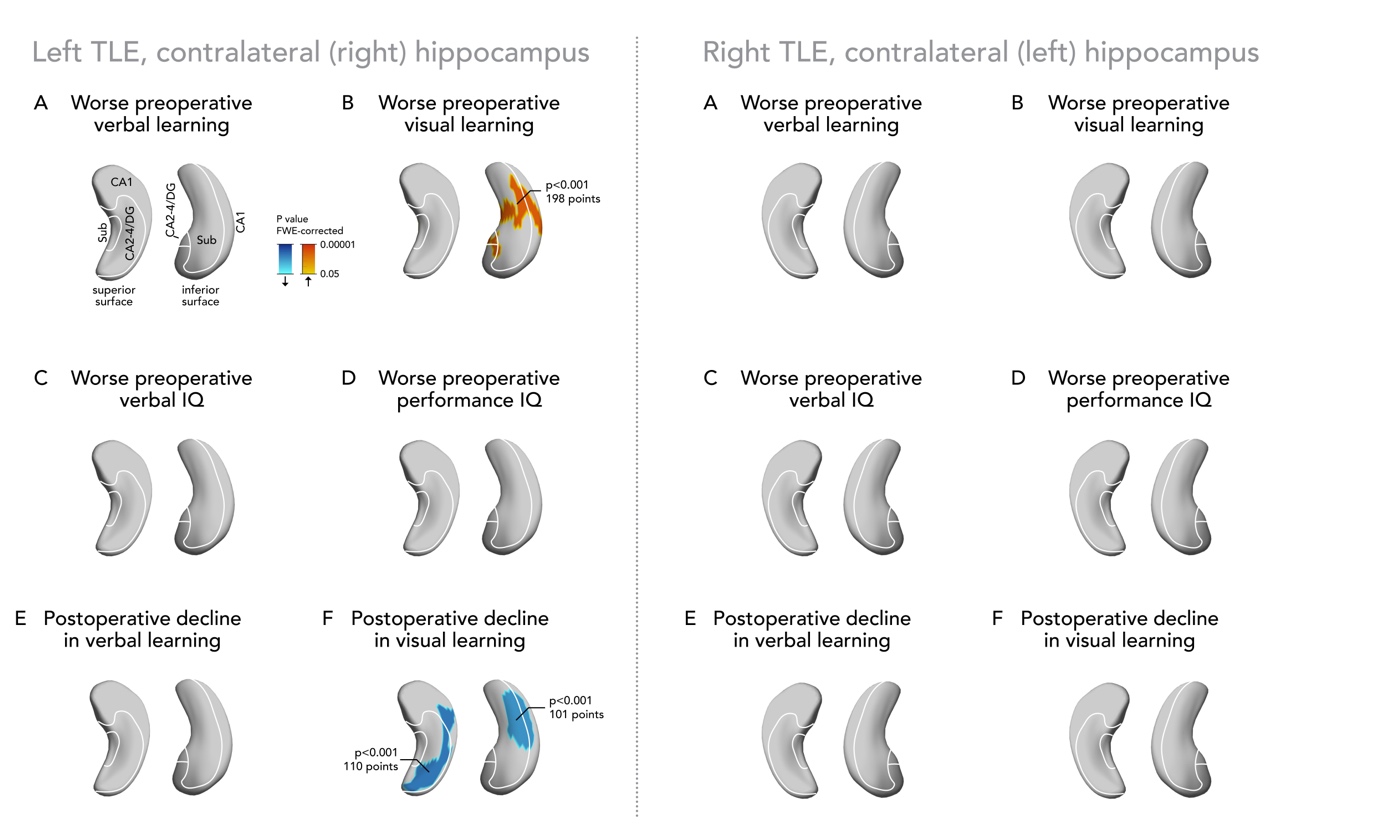


The figure shows accelerated hippocampal morphological changes and their association with neuropsychological findings separately in left and right TLE. We assessed the association of presurgical hippocampal changes with worse preoperative verbal (Panel A) or visual (Panel B) learning, verbal IQ (Panel C), and performance IQ (Panel D). We also assessed the association of postsurgical hippocampal changes with postoperative decline in verbal (Panel E) or visual (Panel F) learning.

Blue clusters indicate significantly less progressive hypertrophy, red colours indicate significantly more progressive hypertrophy. We used linear mixed effects models in presurgical (left, n=10; right, n=5) and postsurgical (left, n=26; right, n=18) TLE patients. Signiﬁcant p values were thresholded to p < 0.05 corrected for multiple comparisons using familywise error (FWE) correction by random ﬁeld theory. The contralateral hippocampi are visualized from a superior and an inferior perspective. An approximation of major hippocampal subregional boundaries is overlaid on hippocampal surfaces.

CA = cornu ammonis; DG = dentate gyrus; FWE = familywise error; IQ = intelligence quotient; Sub = subiculum.

**8. Supplemental Results**

**Supplemental Table 3:** *Coefficient of variation of hippocampal volume*

|  | **Coefficient of variation** | |
| --- | --- | --- |
|  | **Left hippocampus** | **Right hippocampus** |
| **Healthy controls** | 10.9% | 11.3% |
| **Presurgical data** |  |  |
| **Left TLE presurgical** | 28.3%* | 13.5% |
| **Right TLE presurgical** | 15.0% | 28.7%* |
| **Postsurgical data** |  |  |
| **Left TLE postsurgical** | NA | 11.6% |
| **Right TLE postsurgical** | 10.4% | NA |

* high coefficient of variation in the ipsilateral hippocampus due to the co-existence of hippocampal sclerosis and non-sclerotic hippocampi within the same cohort, leading to high variability. TLE = temporal lobe epilepsy.

To summarise, there was low variation in the volumetric measurements of healthy controls and the contralateral hippocampus after surgery. Slightly higher variability was observed in the contralateral hippocampus before surgery. The highest variability was within the ipsilateral hippocampus before surgery, because this group involved individuals with and without hippocampal sclerosis, thus explaining a high variability of volumes.

This demonstrates that there was smaller or comparable data variability in the healthy volunteer cohort than in the patient cohort. The greater the variability the lesser the accuracy for detecting volumetric changes: that the epilepsy group nonetheless showed greater volumetric hippocampal changes shows this is highly unlikely to have resulted from differences in variability, for that would cause an effect in the opposite direction. Thus, our findings cannot be explained by a reduced sensitivity to detect hippocampal volume changes in healthy volunteers as compared to epilepsy patients.

**References**

1. Galovic M, De Tisi J, McEvoy AW, et al. Resective surgery prevents progressive cortical thinning in temporal lobe epilepsy. *Brain*. 2020;143(11):3262-3272. doi:10.1093/brain/awaa284
2. Kogan A, Alpert K, Ambite JL, Marcus DS, Wang L. Northwestern University schizophrenia data sharing for SchizConnect: A longitudinal dataset for large-scale integration. *Neuroimage* 2016; 124: 1196–201
3. Marek K, Jennings D, Lasch S, et al. The Parkinson Progression Marker Initiative (PPMI). *Prog Neurobiol*. 2011;95(4):629-635. doi:10.1016/j.pneurobio.2011.09.005
4. Liu W, Wei D, Chen Q, et al. Longitudinal test-retest neuroimaging data from healthy young adults in southwest China. *Sci Data* 2017; 4: 170017
